# Sleep Duration is Associated with Fruit and Vegetable Intake in Lower Income Adults from the San Francisco Bay Area: A Cross-Sectional Analysis

**DOI:** 10.3390/nu17050848

**Published:** 2025-02-28

**Authors:** Astrid N. Zamora, Michele L. Patel, Maria I. Campero, Dulce M. Garcia, Sofia A. Portillo, Abby C. King

**Affiliations:** 1 Department of Epidemiology and Population Health, Stanford University School of Medicine, Stanford, CA 94304, USA; icampero@stanford.edu (M.I.C.); dgarcia3@stanford.edu (D.M.G.); sofiaaap@stanford.edu (S.A.P.); king@stanford.edu (A.C.K.); 2 Stanford Prevention Research Center, Department of Medicine, Stanford University School of Medicine, Stanford, CA 94304, USA; michele.patel@stanford.edu

**Keywords:** aging adults, dietary intake, fruits and vegetables, fiber, fat, lower income, sex differences, sleep duration, sleep quality

## Abstract

**Background:** Few studies have examined whether sleep is related to dietary intake in aging adults. To address this gap, this study investigated (1) the associations between sleep duration and sleep quality with fruits and vegetables (FV), fiber, and fat intake in lower-income midlife and older adults and (2) sex differences in these relationships. **Methods:** Baseline data from 163 ethnically diverse, lower-income midlife and older adults in the NIH-funded *Steps for Change* trial were analyzed. Dietary intake was assessed using the Block Fruit/Vegetable/Fiber and Fat Intake Screeners, operationalized as weekly servings. Sleep duration (hours per night) and quality were self-reported via the Stanford WELL for Life Scale. Linear regression models assessed the association between sleep duration and FV, fiber, and fat intake, adjusting for potential confounding covariates with separate models for sleep quality. Sex differences were tested using interaction terms, with stratified models also used to explore differences. **Results:** The sample was 73.2% female, with a mean age of 70.5 (SD = 9.7) years (range: 41–99). The mean sleep duration was 6.8 (1.2) hours per night, with 79.2% reporting fairly or very good sleep quality. Mean weekly servings were 24 for FV, 7 for fiber, and 18 for fat. Sleep duration was positively associated with FV intake (β = 2.2; *p* = 0.02). The interaction between duration and sex was marginally significant (*p* = 0.08), with a positive association in males (β = 5.5; *p* = 0.02) but not in females (β = 0.9; *p* = 0.41). No significant associations were found between sleep duration and fiber or fat intake or between sleep quality and any dietary intake outcomes. **Conclusions:** This study found that longer sleep duration was associated with higher FV intake in males but not in females. These findings suggest the possibility of sex differences in the sleep–diet relationship among aging populations that merit further exploration in longitudinal studies.

## 1. Introduction

Consumption of fruits and vegetables (FV) is consistently associated with improved health outcomes across the life course [1,2,3]. In particular, FV intake has been shown to offer enhanced protection against adverse health outcomes and chronic diseases in later life [4,5,6], making it particularly important for midlife adults (defined as ages 41–64) and older adults (defined as ages 65 and older) to meet the recommended intake levels for their age group. However, midlife and older adults from lower-income backgrounds often face significant barriers to accessing and consuming FV [7,8,9]. Barriers may include limited availability, affordability, and accessibility, as well as other socioeconomic challenges. For instance, a study among a sample of adults from Louisiana, United States (US), found that older adults reported consuming less FV than younger adults [7]. A separate study among a South Korean sample revealed that older adults residing in urban settings, particularly those who reported limited food accessibility and affordability, were at increased risk of low vegetable consumption [9].

The cost of locally sourced and in-season FV can be a significant barrier to consumption, particularly among lower-income individuals [10]. This challenge is further compounded for those living in food deserts, where access to fresh produce is limited. In these areas, lower-priced outlets like farmers’ markets or discount grocery stores may be scarce, and transportation barriers further hinder access [10]. Additionally, the affordability of FV is influenced by factors such as the higher cost of organic or specialty produce and the limited availability of affordable outlets in certain regions [11]. Health status may also play a crucial role in FV consumption among older adults. For example, poor oral health or difficulty chewing can discourage the consumption of fresh FV, further exacerbating challenges in meeting dietary recommendations [12].

Given the importance of FV intake for health, emerging research suggests that sleep health patterns may play a role in influencing dietary behaviors, including food choices and FV consumption [13,14,15,16]. While FV intake has been shown to be driven in part by factors such as accessibility and affordability [7,8,9], to the authors’ knowledge, limited studies among aging populations from the US indicate that sleep may also play a role in shaping dietary habits. However, studies have been conducted among the general adult population. For example, a cross-sectional study among adults from the United Kingdom found that long sleepers consumed less FV compared to reference sleepers (i.e., 7–8 h/day) (14). In contrast, short sleepers also consumed less FV than reference sleepers (14). A separate study of young women and men from the US found that better sleep was linked to higher FV intake, with men experiencing higher FV consumption when they had better sleep quality and a shorter time to fall asleep [17]. A potential mechanism underlying the relationship between poor sleep and diet is a disruption of appetite-regulating hormones, leading to increased cravings for high-calorie, low-nutrient foods [18]. However, study inconsistencies, particularly related to differences in measures and populations [18], complicate our understanding of how sleep is related to diet, especially in aging adults.

While studies thus far on sleep and diet in aging adults generally have shown mixed results, these studies have focused on how diet predicts sleep [19,20,21,22,23,24,25] rather than examining how sleep may predict dietary intake. Moreover, few studies have explored the relationship between sleep and FV intake in lower-income populations, who frequently face extreme barriers to accessing FV [26] and often experience sleep health disparities related to their socioeconomic status [27]. Additionally, sex differences in these associations are underexplored despite the importance of understanding how such factors may alter the relationship between sleep and FV intake. For example, females, particularly in midlife and older adulthood, often report poorer sleep quality due to hormonal changes, caregiving responsibilities, and other social determinants of health [28,29,30]. These differences could affect how sleep influences dietary behaviors like FV intake, as females may face unique barriers to healthy eating when experiencing sleep disturbances.

In addition to FV intake, other aspects of diet, such as fiber and fat intake, are essential to consider when examining the relationship between sleep and overall health. Fiber, which has been shown to play a crucial role in metabolic health, digestion, and satiety [31], may also interact with sleep patterns, potentially influencing appetite regulation and energy balance. Similarly, fat intake—particularly the balance of healthy fats—could promote healthy sleep quality and duration through its impact on hormones, inflammation, and overall health [32]. Despite the growing body of research on these dietary components, the links between sleep and fat or fiber intake remain less explored, especially in midlife and older adults facing socioeconomic barriers.

Given the importance of these dietary components, this cross-sectional study aims to explore how sleep duration and quality may be related to the intake of FV, fiber, and fat in a sample of ethnically diverse, lower-income midlife and older adults from the San Francisco Bay Area who were enrolled in the NIH-funded *Steps for Change* trial. A secondary aim was to explore whether these associations varied by sex. Findings from this study could inform whether future nutrition interventions incorporate sleep promotion to improve dietary intake of FV, fiber, and fat.

## 2. Materials and Methods

### 2.1. Study Design and Participants

This cross-sectional secondary analysis utilized baseline data collected between October 2017 and November 2019 from 163 participants enrolled in the NIH-funded 24-month *Steps for Change* trial who had baseline data on both sleep and diet variables [33]. The primary aim of the trial was to evaluate the benefits of adding a neighborhood-focused intervention (*Our Voice*) to the evidence-based Active Living Every Day (ALED) behavioral physical activity program [34]. *Our Voice* is a community-driven initiative designed to empower participants by engaging them in health-related advocacy and environmental change efforts, mainly focusing on health equity and neighborhood improvements at the community level [33]. ALED, on the other hand, is a structured program aimed at promoting physical activity and behavior change through education and self-regulation techniques at the individual level [34].

The trial employed a single-blind, cluster-randomized, controlled parallel design whereby participants were randomized to either *Our Voice* + ALED or ALED only. Outcome assessors were masked to participant intervention assignment, ensuring unbiased assessment of outcomes [33]. It is important to note that this study is based on baseline data and, therefore, the interventions (*Our Voice* + ALED versus ALED only) had not yet begun at the time of the baseline data collection. As a result, the baseline data represents pre-intervention measurements and provides a snapshot of participants’ sleep health and dietary intake behaviors before any programmatic influence.

The trial recruited participants from 10 senior affordable public housing sites across two counties in the San Francisco Bay Area (California, US), a region known for vast economic disparities. Participants represented a lower-income demographic, and the housing sites reflected the area’s ethnic and geographic diversity. Eligible participants were required to be (a) 40 years or older, (b) willing and able to increase their walking time in their neighborhood, (c) able to safely engage in moderate physical activity as assessed by the Physical Activity Readiness Questionnaire (PAR-Q) [35], (d) able to read and understand English or Spanish, and (e) planning to reside in the designated study area for the next 24 months. Ethical approval was obtained from the Stanford University Institutional Review Board, and all participants provided written informed consent prior to enrollment.

### 2.2. Data Collection

#### 2.2.1. Dietary Intake Assessment

Dietary intake was assessed using the validated Block Fruit/Vegetable/Fiber and Fat Screeners [36], which estimate the frequency of consumption of fruits, vegetables, fiber, and fat over the past year. These screeners are reliable and valid tools for rapid dietary assessment, with proven efficacy in multi-ethnic populations [36]. Participants self-reported their intake by considering the following prompt, “*Think about your eating habits over the past year or so. About how often do you eat each of the following foods?*” The Fruit/Vegetable/Fiber Screener includes 10 items, with 7 items focused on the consumption of FV and 3 items on the consumption of fiber-rich foods (i.e., green salads, potatoes, beans). The Fat Intake Screener contains 16 items related to the consumption of fat-rich foods (e.g., fried foods, whole milk, fatty meats). The Fruit/Vegetable/Fiber Screener has 6 response options, which were converted into weekly servings for our analysis: “*Less than once a week*” was assigned a value of 0.5 servings, “*Once a week*” was assigned a value of 1 serving, “*2–3 times a week*” was assigned a value of 2.5 servings, “*4–6 times a week*” was assigned a value of 5 servings, “*Once a day*” was assigned a value of 7 servings, and “*2+ times a day*” was assigned a value of 14 servings per week (for the FV and fiber items). All missing values were left blank, meaning they were not assigned any servings. A similar method was applied to the Fat Intake Screener, with weekly serving values assigned based on the frequency of intake*:“1/month or less”* was assigned a value of 0.25 servings, *“2–3 times a month”* was assigned a value of 0.625 servings, *“1–2 times a week”* was assigned a value of 1.5 servings, *“3–4 times a week”* was assigned a value of 3.5 servings, and *“5+ times a week”* was assigned a value of 5 servings. The weekly servings of the three food groups (FV, fiber, and fat) were then independently summed to obtain a total weekly serving value for each food group per participant. Possible scores ranged from 3.5 to 98 for weekly servings of FV intake, 1.5 to 42 for weekly servings of fiber intake, and 4 to 80 for weekly servings of fat intake. All dietary intake assessment surveys can be found on the NutritionQuest official website (https://www.nutritionquest.com/assessment/ (accessed on 1 November 2024)).

#### 2.2.2. Sleep Duration and Quality

Sleep duration and sleep quality were self-reported by participants using the Stanford WELL for Life Scale [37], which has been utilized in diverse adult populations and includes sleep questions drawn from other psychometrically valid and widely used sleep questionnaires (e.g., the Pittsburgh Sleep Quality Index [38] and the Behavioral Risk Factor Surveillance System (BRFSS) [39]). Sleep duration was captured as the average number of hours participants reported sleeping on a typical night, including decimal values to account for partial hours (e.g., 7.5 h). This information was gathered through the following open-ended question: “*On average, during the last two weeks, how many hours and minutes, not including naps, did you usually sleep during the night?*” [39]. Sleep quality asked participants to respond to the following question: “*During the past two weeks, how would you rate your sleep quality overall?*” with response options on a four-point Likert scale including “Very bad”, “Fairly bad”, “Fairly good”, and “Very good” [38].

#### 2.2.3. Participant Data Collection Procedures

Participant characteristics were collected at baseline, including self-reported sociodemographic factors (age, sex, race/ethnicity, marital status, education, household size, and birthplace), objectively measured health indicators (body mass index [BMI], resting heart rate [pulse], and systolic and diastolic blood pressure), and self-reported health behaviors (physical activity, smoking, and alcohol use). To ensure accurate and consistent measurements of health indicators, trained personnel used validated equipment and followed standardized procedures for anthropometric and blood pressure measurements. Blood pressure, along with heart rate (pulse), was measured using the Omron 7 Series Upper Arm Blood Pressure Monitor (Omron Healthcare, Model BP760N, Lake Forest, IL, USA), with participants instructed to avoid caffeine, heavy physical activity, smoking, and alcohol consumption for at least 30 min prior to measurement. Both systolic and diastolic blood pressure, as well as heart rate, were measured twice, and the average values were used in the analysis. Waist circumference was measured using a Gulick II Tape Measure (North Coast Medical, Model 67020, Morgan Hill, CA, USA), while height and weight were measured with wall-mounted or portable stadiometers and digital scales, respectively. Participants were instructed to wear light clothing and avoid restrictive garments to ensure consistency of measures. Each of the anthropometric measurements (height and weight) was also taken twice, with the average values recorded. Additional attempts were made if necessary to ensure reliable results.

In addition, we obtained information on the self-reported presence of health conditions using a series of questions on a range of common health issues, categorized into the following health conditions: cancer (excluding minor skin cancers), cardiovascular (CVD) (e.g., heart conditions such as heart attack, heart surgery, irregular rhythm, congestive heart failure, coronary artery disease, or high blood pressure), metabolic (i.e., diabetes, high cholesterol, hypothyroidism, hyperthyroidism), musculoskeletal (e.g., gout, osteoarthritis, rheumatoid arthritis, osteoporosis), neurological (e.g., stroke, head injury, narcolepsy, multiple sclerosis, dementia, Parkinson’s disease), psychiatric (e.g., anxiety, severe depression, behavioral mood disorders, schizophrenia), pulmonary (e.g., COPD, asthma, emphysema, sleep apnea), and sensory abnormalities (e.g., visual, hearing impairments, peripheral neuropathy). Each participant was asked whether they had been diagnosed with any of the conditions listed, with three possible responses: “Yes,” “No,” or “Don’t Know/Refused/Missing”. The “Don’t Know/Refused/Missing” category was combined into one category for analytical purposes.

Participants’ race/ethnicity was categorized into two groups based on self-report: “Non-Hispanic White” [NH-White] and “Minority racial/ethnic groups”, which included individuals identifying as Latino/a, African American or Black, Asian, or Multiracial. This two-category grouping was adopted because most existing literature comparing health outcomes and behaviors across racial/ethnic groups has primarily focused on differences between NH-White individuals and minority racial/ethnic groups, as these comparisons often reveal significant disparities [40,41]. Birthplace was recorded as either “US-born” or “Not US-born”. Household size was defined as the total number of individuals living in the participant’s household. BMI was calculated using participants’ weight and height, with measurements taken in person by trained clinical staff using a calibrated scale and stadiometer. Resting heart rate and blood pressure were measured twice by trained clinical staff, and the average of the two readings was used in the analysis. Total physical activity (minutes per week) was self-reported using the Community Healthy Activities Model Program for Seniors (CHAMPS) questionnaire [42]—a validated questionnaire designed to capture weekly minutes of different types and intensities of PA among midlife and older adults. Smoking status was determined based on whether the participant was a current smoker (Yes/No); this binary categorization ensured that our analysis centered on the present risk associated with ongoing smoking behavior. Alcohol intake was assessed by the weekly number of alcoholic beverages reported over the past 12 months.

Adjusted statistical models incorporated the following covariates based on prior knowledge of their associations with sleep and dietary intake: age, sex, education, total physical activity, household size, and presence of cardiovascular health condition(s*)* [17,32,43,44,45].

### 2.3. Statistical Analysis

First, descriptive statistics were computed to characterize the study sample in terms of sociodemographic and health-related variables. Because our secondary aim focused on examining sex differences in the association between sleep predictors and dietary outcomes, we examined differences in baseline characteristics according to sex (male versus female). The chi-square test or Fisher’s exact test was used to analyze differences in all binary and categorical variables. Fisher’s exact test was employed in instances where the expected cell counts were low (typically less than 5) or when the sample size was small, ensuring more accurate results in those situations. T-tests were used to compare continuous variables between groups. Continuous variables are reported as means with standard deviations, while categorical variables are summarized using frequencies and percentages. Normality assumptions were checked for continuous variables, and equal variances were tested where applicable for *t*-tests.

To assess the primary research question, separate linear regression models were used to examine the relationship between sleep duration—measured in hours per night—(the independent variable) and weekly servings of FV, fiber, and dietary fat (the dependent variables). Separate models were constructed to explore the association between sleep quality and each dietary outcome. All models were adjusted for the aforementioned confounders. For models with sleep duration as the independent variable, we adjusted for sleep quality due to the correlation between the two sleep variables.

Sex differences in the associations between the sleep variables and dietary intake were examined using interaction terms (sex × sleep duration and sex × sleep quality) in separate regression models. Additionally, stratified models were used to explore these differences in more detail by separately analyzing the relationships between sleep variables and dietary intake for males and females.

All statistical analyses were performed using SAS 9.4 (Cary, NC, USA), with a significance level of *p* < 0.05 for all tests and *p* < 0.10 used to assess marginal significance, given the exploratory nature of the analysis and the moderate sample size.

## 3. Results

### 3.1. Participant Characteristics

Table 1 presents baseline participant characteristics of the total analytic sample and the sample stratified by sex. Overall, the mean (SD) age of the participants was 70.5 (9.7) years (range: 41–99), with 73.0% of the sample being female and 76.7% identifying as non-Hispanic White. Significant differences were observed between males and females according to marital status (*p* ≤ 0.001), household income (*p* = 0.003), and household size (*p* = 0.04), with more females being separated or divorced, having a lower income, and living in smaller households. CVD conditions were present among 47.2% of the overall analytic sample. When stratified by sex, there was a higher proportion of males (27/44) with CVD than females (50/119) (*p* = 0.03). The mean (SD) sleep duration was 6.8 (1.2) hours per night. In terms of sleep quality, 79.2% reported “fairly good” or “very good” sleep, including 83% of females and 68% of males. Neither sleep variable differed by sex. The mean (SD) weekly servings of the dietary intake outcomes included the following: fruit/vegetable intake of 24.3 (13.7), fiber intake of 6.6 (5.2), and fat intake of 18.2 (7.2) servings, with no differences by sex.

### 3.2. Associations Between Sleep Duration and Quality with Dietary Intake Outcomes

In the overall analysis, a significant positive association was observed between sleep duration and FV intake. Specifically, each 1 h increase in sleep duration was associated with an increase of 2.2 weekly servings of FV (β = 2.2, 95% CI: 0.2, 4.1; *p* = 0.02) in the fully adjusted model (Table 2). No significant associations were found between sleep duration and fiber or fat intake (fiber: *p* = 0.75, fat: *p* = 0.47). Sleep quality was not significantly associated with any dietary intake outcome in either the unadjusted or the fully adjusted model. Specifically, the relationships between sleep quality and FV intake (β = −0.5, *p* = 0.75), fiber intake (β = −0.5, *p* = 0.46), and fat intake (β = −0.8, *p* = 0.39) were all non-significant in the fully adjusted model.

### 3.3. Sex-Stratified Associations Between Sleep Duration and Quality with Dietary Intake Outcomes

In fully adjusted models, sex differences were examined using interaction terms, revealing a marginally significant association between sleep duration and FV intake (Table 3). Specifically, for men, the interaction between core sleep hours and sex was significant (β = 3.4, 95% CI: −0.5, 7.2; *p* = 0.08), suggesting a positive association between sleep duration and FV intake, though marginally significant. To further explore sex differences, fully adjusted sex-stratified models were conducted. In men, a 1 h increase in sleep duration was associated with an increase of 5.5 weekly servings of FV (β = 5.5, 95% CI: 0.9, 10.1; *p* = 0.02), whereas no such association was found in females (β = 0.9, 95% CI: −1.3, 3.0; *p* = 0.41). No significant associations were observed between sleep duration and fiber or fat intake for either sex. Additionally, there were no significant sex differences in the associations between sleep quality and any dietary intake outcomes.

## 4. Discussion

This study examined the relationships between two critical sleep health measures—sleep duration and sleep quality—and dietary intake of FV, fiber, and fat in a racially/ethnically diverse, lower-income sample of midlife and older adults. Our findings indicate that sleep duration was positively associated with increased FV intake across the overall sample. Notably, however, sex differences were evident, with this relationship being significant only for men. Meanwhile, sleep quality showed no significant associations with any of the dietary outcomes in the overall sample or in the sex-stratified analysis. These null findings should be interpreted with caution, as they may be influenced by the study design and sample characteristics, including the small sample sizes in specific subgroups.

The link between longer sleep duration and greater intake of FV aligns with a prior study conducted among US adults and veterans [46] and among adolescents [47]. Those studies found that shorter sleep duration was associated with low intake of FV [46], suggesting that this relationship between sleep duration and FV intake may persist across different populations and at different stages across the life course. Moreover, to the best of our knowledge, limited to no studies have examined the link between FV, fat, or fiber intake and sleep duration or sleep quality in older adults from low-income backgrounds. However, a few cross-sectional studies have explored the bidirectional associations between these factors in a broader older adult population. For example, a study of older adults (aged ≥60 years) from Spain found that adherence to a Mediterranean diet pattern was associated with a lower risk of changes in sleep duration and with better sleep quality [23]. Additionally, a study among Chinese older adults found a positive association between good sleep quality, appropriate sleep duration, and frequent FV intake [48]. These findings underscore the potential role of sleep in influencing dietary behaviors among older adults. In addition to the associations observed between sleep duration and the consumption of FV, it is important to acknowledge the potential impact of other dietary factors on sleep, such as caffeine. Caffeine, commonly found in coffee, tea, and certain sodas, has been well documented as a significant factor influencing sleep patterns [49]. While our study did not include caffeine intake, it is plausible that caffeine consumption may have confounded or moderated the observed associations between sleep and dietary intake of FV. Future research should assess detailed caffeine intake, and macronutrient, micronutrient, and other food groups will be valuable in expanding our understanding of how various dietary components, beyond FV, interact with sleep and influence overall health outcomes.

While the exact underlying mechanisms remain unclear, studies suggest that sleep duration influences appetite and self-regulation, both of which could mediate its effects on dietary behaviors. First, shorter sleep duration may increase appetite by elevating an appetite-stimulating hormone (ghrelin) and/or reducing an appetite-suppressing hormone (leptin) [50]. This imbalance may lead to heightened appetite and cravings for energy-dense, nutrient-poor foods [50]. On the other hand, sufficient sleep helps restore normal levels of these hormones, reducing hunger and promoting healthier food choices, including FV intake. Elevated ghrelin levels have specifically been associated with increased cravings for carbohydrate-rich and starchy foods [51], which could indirectly reduce the consumption of FV.

Second, adequate sleep plays a critical role in enhancing self-regulation. Sleep deprivation impairs cognitive function and decision-making, often leading to impulsive food choices and a preference for high-calorie, low-nutrient foods [52]. By restoring cognitive function and decision-making capacity, longer sleep duration may improve self-regulation, making it easier for individuals to resist unhealthy food cravings and opt for healthier, nutrient-dense foods such as FV. These mechanisms could explain the observed positive relationship between longer sleep duration and greater FV consumption.

The World Health Organization’s “5 A Day” campaign recommends consuming at least five servings of FV per day, or approximately 35 servings per week, to help reduce the risk of severe health conditions like heart disease [53]. In this study, participants reported a mean intake of 24.3 servings per week, which is below the recommended amount but higher than the 17.5 weekly servings reported for US adults in 2022 [54]. This gap underscores the need for strategies aimed at increasing regular FV consumption in populations of midlife and older adults like ours. If future longitudinal studies establish the causal link between sleep duration and FV intake, it could have important implications for public health interventions focused on improving dietary behaviors.

In examining sex differences, we found that longer sleep duration was linked to higher FV intake in males only. While there are limited studies on these associations among older adults, this finding is consistent with a previous longitudinal study of adults in South Africa (ages 40 to ≥80), which found that higher FV intake was associated with poorer sleep quality among men but not women [55]. A separate study among young adult men found a similar association, in that those who took longer to fall asleep consumed significantly fewer servings of FV compared to those who fell asleep more quickly [17]. Further research is needed to understand better how sex and age may interact to influence the relationship between different markers of sleep and FV intake. We posit that several factors may help explain the observed sex differences in the present study. First, psychosocial factors may influence this relationship. For example, females, particularly older females, often report higher caregiving responsibilities, which can lead to disrupted sleep patterns, lower sleep quality [56], and shifts in dietary priorities [57]. Caregiving roles may limit time and energy for meal planning and preparation, potentially leading to less frequent consumption of FV. These factors could diminish the positive impact of sleep on diet in females. In contrast, males may experience fewer competing responsibilities [58], leading to a more direct relationship between sleep duration and FV intake. Given the observed sex differences, future longitudinal research with repeated measures is needed to determine whether the relationship between sleep duration and FV intake remains consistent over time. Moreover, studies examining this relationship in different populations of males and females and among those with poorer sleep could provide further insights into how sex-specific factors may shape dietary behaviors, helping to clarify whether these associations persist in diverse contexts.

In this sample of aging adults, sleep duration was generally adequate and had little variability, with a mean of 6.8 and SD of 1.2 h per night. While no significant associations were observed between sleep duration and fiber or fat intake—contrasting with some studies that observed broader dietary associations [59,60]—this discrepancy may be due to differences in the specific food groups measured by the instruments being used or other unaccounted factors. Further, the relation between sleep duration and dietary intake may be easier to detect in samples with greater variability. In addition, sleep quality was good, on average, with 83% of females and 68% of males rating it as fairly or very good. It is possible that the link between sleep quality and dietary intake may be more pronounced in populations with poorer sleep quality.

## 5. Strengths and Limitations

Our study has several strengths, including its focus on a lower-income, racially and ethnically diverse sample of midlife and older adults living in affordable housing communities, a group often underrepresented in sleep and dietary research [59,61]. By including both males and females across midlife and older adulthood, we were able to explore sex differences in the sleep–diet relationship, an area of research that remains underexplored [59,61]. Additionally, the inclusion of a diverse sample enhances the external validity of the findings, providing valuable insights into health behaviors in underrepresented populations. The use of validated surveys for dietary intake [36] further strengthens the reliability of the data. While the cross-sectional design limits causal inferences, it offers valuable preliminary data that could inform future longitudinal studies examining these associations. Other study limitations include the use of self-reported questionnaire data on both sleep and dietary factors, which may introduce bias, such as under- or over-reporting of dietary intake [62] and sleep duration [63]. The broad age range of participants (41–99 years) may have introduced variability in the consumption of FV. While we adjusted for age in regression models to account for its potential confounding effect, future longitudinal studies could explore how age may serve as a moderator of the effect of diet on sleep. In addition, the small sample size for males (n = 44) may have reduced statistical power to detect additional sex-specific associations. Future research should explore these relationships with larger sample sizes, longitudinal designs, and data collected across different time periods to clarify the stability and causal direction of these associations. We acknowledge that relying on self-reported presence of health conditions may have introduced bias, and that additional, more objective data on the presence and severity could improve our understanding of how these factors influence sleep and dietary behaviors. Another limitation of this study is the potential impact of seasonal fluctuations in food availability on participants’ dietary intake. Although the Block questionnaires ask participants to reflect on their typical dietary patterns over the past year, this approach does not capture precise seasonal variations in food consumption. Given that the availability of fruits, vegetables, and other foods may fluctuate with the seasons, it is possible that seasonal shifts in food intake were not fully accounted for in this study. Future studies may benefit from incorporating more frequent or seasonal dietary assessments to better understand how seasonal changes in food availability and consumption may affect dietary behaviors. While the study sample was diverse, with more than a quarter of participants self-identifying as racial/ethnic minorities and the majority falling into the lower-income category for this region [64,65], it may not be fully representative of other lower-income aging populations in different geographic regions of the US. This could limit the generalizability of the findings. Future work should consider including a broader range of geographic locations to increase the external validity of the results. Finally, while the baseline data for this study were collected prior to the COVID-19 pandemic, it is important to recognize that the pandemic has had a profound impact on health behaviors, including sleep [66] and dietary patterns [67]; future studies should examine whether these findings are generalizable in a post-COVID-19 pandemic era.

## 6. Conclusions

In conclusion, this cross-sectional study found that longer sleep duration was positively associated with FV intake, particularly among males. While these findings provide valuable insights, further prospective studies with larger samples and greater variability in sleep metrics are needed to confirm these associations and explore potential causal relationships. Should these results be replicated, they could inform future public health strategies aimed at improving both sleep duration and dietary habits as complementary health behaviors that contribute to overall well-being in aging populations. Future research should also focus on understanding the mechanisms behind sex differences and how these factors can be addressed to promote healthier sleep and eating patterns in older adults.

## Figures and Tables

**Table 1:** Baseline participant characteristics.

Participant Characteristics	Entire Sample (n = 163)	Female (n = 119)	Male (n = 44)
Sociodemographic factors			
Age, years [mean (SD)]	70.5 (9.7)	70.2 (9.7)	71.3 (9.7)
*p*		0.50	
Sex, N (%)			
Male	44 (27.0)	0 (0)	44 (27.0)
Female	119 (73.0)	119 (73.0)	0 (0)
*p*		n/a ^2^	
Race/ethnicity, N (%)			
Non-Hispanic White	125 (76.7)	89 (54.6)	36 (22.1)
Minority racial/ethnic groups ^1^	38 (23.3)	30 (18.4)	8 (4.9)
*p*		0.34	
Marital status, N (%)			
Married	66 (40.5)	37 (22.7)	29 (17.8)
Separated/divorced	53 (32.5)	48 (29.5)	5 (3.1)
Widowed	31 (19.0)	23 (14.1)	8 (4.9)
Never married	7 (4.3)	6 (3.7)	1 (0.61)
Other	5 (3.7)	5 (3.1)	1 (0.61)
*p*		<0.001	
Education level, N (%)			
Elementary school	3 (1.8)	2 (1.2)	1 (0.61)
High school/equivalent	28 (17.2)	22 (13.5)	6 (3.7)
College graduate	81 (49.7)	64 (39.3)	17 (10.4)
Postgraduate	51 (31.3)	31 (19.0)	20 (12.3)
*p*		0.11	
Income, N (%)			
<USD 15,000	11 (6.8)	9 (5.5)	2 (1.2)
USD 15,000 to USD 34,999	16 (9.8)	14 (8.6)	2 (1.2)
USD 35,000 to USD 74,999	32 (19.6)	19 (11.7)	13 (8.0)
USD 75,000 or greater	56 (34.4)	34 (20.9)	22 (13.5)
Don’t know/refused	48 (29.5)	43 (26.4)	5 (3.1)
*p*		0.003	
Household size [mean (SD)]	1.9 (1.1)	1.8 (1.1)	2.2 (1.1)
*p*		0.04	
US-born, N (%)			
Yes	107 (65.6)	79 (48.5)	28 (17.2)
No	56 (34.4)	40 (24.5)	16 (9.8)
*p*		0.74	
Health-related factors			
Body mass index, kg/m^2^ [mean (SD)]	29.2 (6.1)	29.1 (6.3)s	29.5 (5.6)
*p*		0.70	
Resting heart rate, bpm [mean (SD)]	69.2 (11.2)	69.5 (11.2)	68.4 (11.3)
*p*		0.58	
Systolic blood pressure, mmHg [mean (SD)]	121.8 (15.0)	121.9 (14.9)	121.6 (15.4)
*p*		0.89	
Diastolic blood pressure, mmHg [mean (SD)]	76.3 (9.0)	77.0 (9.0)	74.3 (8.9)
*p*		0.08	
Presence of cancer			
No	137 (85.1)	100 (61.4)	37 (22.7)
Yes	25 (15.3)	18 (11.0)	7 (4.3)
Don’t know/refused/missing	1 (0.6)	1 (0.6)	0 (0)
*p*		1.0	
Presence of CVD condition			
No	86 (52.8)	69 (42.3)	17 (10.4)
Yes	77 (47.2)	50 (30.7)	27 (16.6)
Don’t know/refused/missing	0 (0)	0 (0)	0 (0)
		0.03	
Presence of metabolic condition			
No	81 (49.7)	65 (39.9)	16 (9.8)
Yes	80 (49.1)	53 (32.5)	27 (16.6)
Don’t know/refused/missing	2 (1.2)	1 (0.6)	1 (0.6)
		0.06	
Presence of musculoskeletal condition			
No	83 (50.9)	58 (35.6)	25 (15.3)
Yes	74 (45.4)	57 (35.0)	17 (10.4)
Don’t know/refused/missing	6 (3.7)	4 (2.5)	2 (1.2)
		0.55	
Presence of neurological condition			
No	150 (92.0)	108 (66.3)	42 (25.8)
Yes	12 (7.4)	10 (6.1)	2 (1.2)
Don’t know/refused/missing	1 (0.6)	1 (0.6)	0 (0)
		0.64	
Presence of psychiatric condition			
No	129 (79.1)	96 (58.9)	33 (20.3)
Yes	32 (19.7)	23 (14.1)	9 (5.5)
Don’t know/refused/missing	2 (1.2)	0 (0)	2 (1.2)
		0.11	
Presence of pulmonary condition			
No	133 (81.6)	96 (58.9)	37 (22.7)
Yes	30 (18.4)	23 (14.1)	7 (4.3)
Don’t know/refused/missing	0 (0)	0 (0)	0 (0)
		0.82	
Presence of sensory abnormalities			
No	0 (0)	0 (0)	0 (0)
Yes	0 (0)	0 (0)	0 (0)
Don’t know/refused/missing	163 (100.0)	119 (73.0)	44 (27.0)
		n/a ^3^	
Sleep-related factors			
Sleep duration, hours per night [mean (SD)]	6.8 (1.2)	6.9 (1.2)	6.7 (1.3)
*p*		0.47	
Sleep quality, N (%)			
Very bad	3 (1.8)	1 (0.61)	2 (1.2)
Fairly bad	31 (19.0)	19 (11.7)	12 (7.4)
Fairly good	102 (62.6)	79 (48.5)	23 (14.1)
Very good	27 (16.6)	20 (12.3)	7 (4.3)
*p*		0.13	
Dietary factors			
Fruit/vegetables, servings/wk [mean (SD)]	24.3 (13.7)	23.6 (12.6)	26.1 (16.3)
*p*		0.30	
Fiber, servings/wk [mean (SD)]	6.6 (5.2)	6.4 (4.6)	7.3 (6.8)
*p*		0.31	
Fat, servings/wk [mean (SD)]	18.2 (7.2)	17.7 (7.1)	19.5 (7.4)
*p*		0.17	
Other health behaviors			
Alcoholic, drinks/wk [mean (SD)]	1.6 (3.5)	1.4 (2.7)	2.3 (5.1)
*p*		0.12	
Smoking status, N (%)			
No	161 (98.8)	118 (72.4)	43 (26.4)
Yes	2 (1.2)	1 (0.61)	1 (0.61)
*p*		0.46	
Total physical activity, min/wk [mean (SD)]	594.6 (356.9)	601.2 (341.3)	576.9 (399.6)
*p*		0.70	

^1^ Minority racial/ethnic groups included participants that identified as Latino/a, African American/Black, Asian, or Multiracial; ^2^ *p*-value not applicable due to stratification by sex; ^3^ *p*-value not applicable due to no variation in responses across participants; abbreviations: bpm: beats per minute; wk: week; h: hours; SD: standard deviation.

**Table 2:** Associations between sleep duration and quality with dietary intake outcomes.

	Dietary Intake Outcomes	Model 1: Unadjusted β (95% CI)	Model 2: Fully Adjusted Model ^1^ β (95% CI)
Sleep duration			
	Fruits and vegetables (servings/wk)	2.0 (0.3, 3.8); *p* = 0.02 **	2.2 (0.2, 4.1); *p* = 0.02 **
	Fiber intake (servings/wk)	0.11 (−0.6, 0.8); *p* = 0.75	0.2 (−0.6, 0.9); *p* = 0.67
	Fat intake (servings/wk)	−0.08 (−1.0, 0.8); *p* = 0.86	0.2 (−0.9, 1.2); *p* = 0.75
Sleep quality			
	Fruits and vegetables (servings/wk)	1.0 (−2.2, 4.3); *p* = 0.53	0.2 (−0.9, 1.2); *p* = 0.75
	Fiber intake (servings/wk)	−0.23 (−1.5, 1.0); *p* = 0.71	−0.5 (−2.0, 0.9); *p* = 0.45
	Fat intake (servings/wk)	−0.63 (−2.3, 1.1); *p* = 0.47	−0.9 (−2.8, 1.0); *p* = 0.37

^1^ Model 2 adjusted for age, sex, education, total physical activity, presence of CVD health condition(s), and household size, and for models with sleep duration as the predictor, we adjusted for sleep quality due to the correlation between the two sleep variables. In models with sleep quality as a predictor, we adjusted for the same covariates in addition to sleep duration. Notes: ** denotes < 0.05.

**Table 3:** Sex differences in the associations between sleep duration and quality with dietary intake outcomes.

	Dietary Intake Outcomes	Interaction Model: Sleep Predictors × Sex ^1,2^ β (95% CI)	Stratified Model: Male ^1^ β (95% CI)	Stratified Model: Female ^1^ β (95% CI)
Sleep duration				
	Fruits and vegetables (servings/wk)	3.4 (−0.5, 7.2); *p* = 0.08 *	5.6 (1.0, 10.2); *p* = 0.02 **	0.9 (−1.3, 3.0); *p* = 0.42
	Fiber intake (servings/wk)	0.7 (−0.6, 2.0); *p* = 0.30	0.5 (−1.6, 2.5); *p* = 0.65	0.01 (−0.7, 0.8); *p* = 0.96
	Fat intake (servings/wk)	−0.3 (−2.1, 1.5); *p* = 0.74	−0.8 (−3.1, 1.5); *p* = 0.50	0.5 (−0.6, 1.7); *p* = 0.37
Sleep quality				
	Fruits and vegetables (servings/wk)	−1.2 (−6.9, 4.6); *p* = 0.69	−5.2 (−13.2, 2.7); *p* = 0.19	0.5 (−3.9, 4.8); *p* = 0.83
	Fiber intake (servings/wk)	0.7 (−1.5, 2.9); *p* = 0.50	0.5 (−3.0, 4.1); *p* = 0.75	−1.1 (−2.6, 0.4); *p* = 0.15
	Fat intake (servings/wk)	0.1 (−3.0, 3.1); *p* = 0.95	0.4 (−3.6, 4.5); *p* = 0.83	−1.9 (−4.2, 0.5); *p* = 0.11

^1^ Results from models adjusted for age, sex, education, total physical activity, presence of CVD health condition(s), and household size; ^2^ β (95% CI) from the interaction model is for male participants. Notes: * denotes < 0.10; ** denotes < 0.05.

## Data Availability

Deidentified datasets used in this study will be made available upon reasonable request to the corresponding author (astridz@stanford.edu) due to privacy reasons.
